# Interaction of body mass index and hemoglobin concentration on blood pressure among pregnant women in Guangxi, China

**DOI:** 10.1186/1471-2458-14-474

**Published:** 2014-05-20

**Authors:** Qiuan Zhong, Jiangyan Xu, Yingquan Long, Yingying Deng, Jinlan Hu, Xiaofei Li, Xiaoqiang Qiu

**Affiliations:** 1 Department of Epidemiology, Guangxi Medical University School of Public Health, 22 Shuangyong Road, Nanning, Guangxi 530021, China; 2 Department of Pediatrics, Nanning Maternal and Child Health Hospital, Nanning, China; 3 Department of Comprehensive Laboratory, Guigang Entry-Exit Inspection and Quarantine Bureau, Guigang, China

**Keywords:** Body mass index, Hemoglobin, Blood pressure, Interaction, Pregnancy

## Abstract

**Background:**

Body mass index (BMI) and hemoglobin (Hb) are positively associated with hypertensive disorders among pregnant women. The aim of this study was to estimate a potential interaction between high BMI and high Hb concentrations on systolic blood pressure (SBP) and diastolic blood pressure (DBP) in pregnancy.

**Methods:**

We recruited 4497 single-birth women aged 18–43 years who received routine antenatal care at three hospitals of Guigang, Guangxi, China, from December 2007 to January 2011. Of 4497 participants, 3472 women were in the first trimester, with following up, 2986 women and 2261 women were left in the second and third trimester, respectively. Clinical data were derived from medical records of each woman. We used multivariable linear regression, by trimesters of pregnancy, to evaluate the associations of high BMI and high Hb concentrations with SBP and DBP according to cross-sectional design.

**Results:**

In multivariable analyses, BMI was positively associated with SBP throughout all trimesters, but the corresponding association for Hb concentrations only in the first trimester, whereas both BMI and Hb concentrations were positively associated with DBP in the first and third trimesters. After full adjustment for confounding, the average differences in SBP and DBP comparing women with high BMI and high Hb to those with non-high BMI and non-high Hb were 2.9 mmHg (95% CI: 0.8 to 5.0 mmHg) and 3.9 mmHg (95% CI: 1.5 to 6.3 mmHg) in the first trimester, 2.6 mmHg (95% CI: 0.4 to 4.8 mmHg) and 1.5 mmHg (95% CI: -1.3 to 4.3 mmHg) in the second trimester, and 4.8 mmHg (95% CI: 2.3 to 7.4 mmHg) and 5.7 mmHg (95% CI: 3.2 to 8.3 mmHg) in the third trimester, respectively. With respect to the interaction, significant combined effects between high BMI and high Hb were confirmed on SBP (*P* = 0.02) and DBP (*P* = 0.004) in the third trimester, and the amount of interaction on SBP and DBP were 2.0 mmHg (95% CI: 0.1 to 3.9 mmHg) and 2.3 mmHg (95% CI: 0.4 to 4.3 mmHg), respectively.

**Conclusion:**

Our findings suggest that high BMI and high Hb concentrations may have a synergistic effect on blood pressure in late stage of pregnancy.

## Background

High blood pressure during pregnancy contributes to the risks of not only adverse neonatal outcomes but also maternal deaths [1,2]. Although the etiology of hypertensive disorders during pregnancy is not yet completely clear, maternal anthropometric measure such as high body mass index (BMI) in pregnancy has been reported to increase the risk of pregnancy-induced hypertension (PIH) or preeclampsia [3-6]. Similarly, several studies have shown that maternal hemoglobin (Hb) levels were positively associated with PIH [7-9]. Moreover, a review suggests that higher than normal hemoglobin concentrations should be regarded as an indicator of possible pregnancy complications [10]. Some of the above findings [4,5,7], however, may be limited because of small sample size or insufficient gestational weeks.

The mechanisms underlying the positive association of Hb levels with pregnancy blood pressure are incompletely understood, but previous evidences suggested that elevated Hb levels might impact hypertensive disorders in pregnant [7,10,11] as well as non-pregnant women [12] via hemoconcentration or increased blood viscosity, which is generally associated with both overall adiposity and abdominal adiposity [13]. Further, a population-based study, particularly on the association between Hb levels and BMI during pregnancy, showed that Hb levels were significantly associated with BMI in 561 pregnant women [14]. However, less is known about the combined effect of BMI and Hb levels on blood pressure in pregnancy. Thus, in this large sample study, we further evaluated the potential interaction between high BMI and high Hb concentrations on systolic blood pressure (SBP) and diastolic blood pressure (DBP) in all trimesters of pregnancy.

## Methods

### Study population

First, we categorized all hospitals that can provide a routine antenatal care into three stratifications including primary, secondary, and tertiary hospitals in Guigang, Guangxi Zhuang Autonomous Region, China, then randomly selected one hospital from each stratification using cluster sampling. The present study was embedded in the antenatal care at three hospitals during December 2007 to January 2011. A total of 5701 healthy women aged ≥18 years with singleton pregnancies who attended their the first antenatal care at gestational weeks 10 to 16 were accumulatively enrolled, we excluded 41 in vitro fertilization and 1163 with incomplete data. Finally, 4497 eligible women aged 18–43 years were left to follow up with the routine antenatal visits. Gestational age was based on the first day of the last menstrual period, for 1169 women (26%) with neither a known first day of the last menstrual period nor a regular menstrual cycle, the ultrasound estimate was used at the first antenatal visit. Pregnancy was classified into the first, second, and third trimesters using gestational age: <14, 14–27, and 28–42 weeks, respectively.

**Figure 1:**
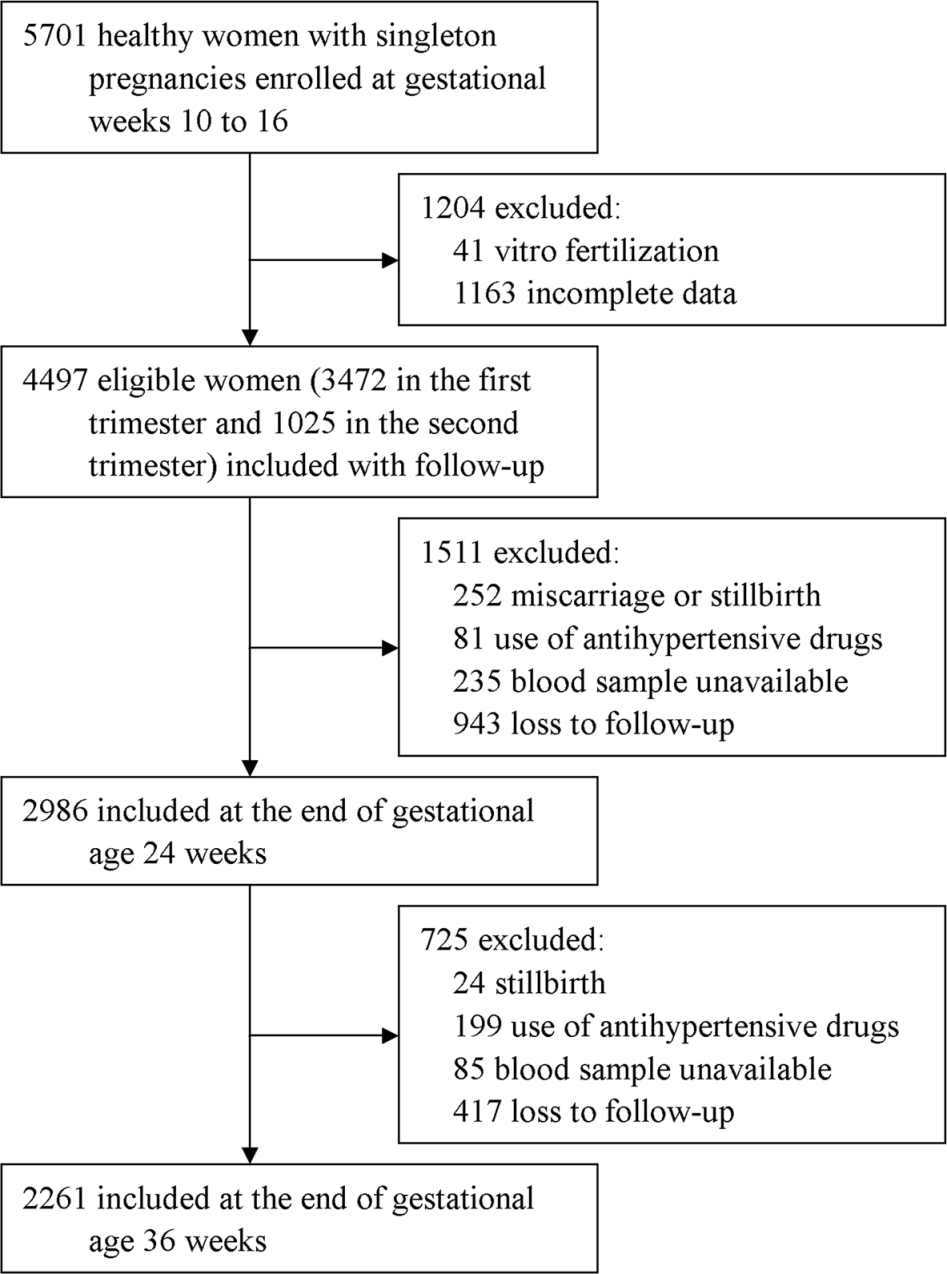
**Flow diagram of study participants throughout pregnancy.** 3472, 2986, and 2261 at each time point represent the number of women with complete data including body mass index (BMI), blood pressure, and blood samples as well as basic characteristics in the first, second and third trimesters, respectively.

The follow-up of study participants throughout pregnancy is shown in Figure 1. Of 4497 participants at study entry, 3472 women were in the first trimester, 1025 women were in the second trimester. With following up, 2986 women and 2261 women were left at the end of gestational weeks 24 and 36, respectively. All eligible participants provided written informed consent, the Guangxi Medical University Institutional Review Board approved the present protocols.

### Data collection and laboratory measures

The three hospitals performed the same antenatal examination, laboratory procedure, and quality control. At the first antenatal visit, baseline information on maternal age, ethnicity, education, family income, residence, gestational age, parity, and folic acid supplement was based on interview questionnaires, anthropometric measures (e.g. body weight and height) and blood pressure were recorded through physical examinations. Blood pressure readings and body weight were prospectively recorded from the first to the last antenatal visit. Blood pressure was measured with Omron HEM 907 IntelliSense professional digital blood pressure monitor (Omron Healthcare Ltd., Dalian, China) using an averaging mode in a sitting position. After at least 5 minutes of rest, an appropriately sized upper arm cuff was applied to the right arm, the mean value of two blood pressure readings with a pause of 60 seconds between each measurement was recorded.

Fasting blood was repeatedly collected at the first antenatal visit that ranged from gestational weeks 10 to 16, around gestational weeks 20, 24, 32, and 36. All samples were performed using the standard protocol for clinical laboratory tests. Hb concentration value in whole blood was performed with Beckman Coulter AcT 5 Diff hematology analyzer (Beckman Coulter Inc., Fullerton, USA), and was determined via cyanmethhemoglobin method. The interassay coefficients of variation for Hb concentrations ranged from 3.1% to 8.9% throughout the study period. Serum total cholesterol, serum triglycerides, and plasma glucose were measured using Hitachi 7170A or 7020 full-automatic biochemical analyzer (Hitachi Ltd., Tokyo, Japan).

### Definitions of variables

BMI was calculated by dividing weight in kilograms by standing height that was measured at enrollment in meters squared (kg/m^2^). High BMI was defined as being more than or equal to the 90th percentile cutoff of BMI. High Hb concentration was defined as an Hb level of ≥13.0 g/dL [15]. Family income was the total combined family income including wages, salaries, self-employment, and the other source during the last 12 months. Urban residence was defined as urban districts, central or fringe areas of city or town with a population density higher than 1500/km^2^. Parity was the numbers of times woman has given birth including stillbirths. Folic acid supplement was defined as women took a daily supplement of folic acid during 3 months before pregnancy to the first antenatal visit. Diabetes mellitus was defined as a fasting glucose ≥126 mg/dL, a non-fasting glucose ≥200 mg/dL, or a self-report physician diagnosis, or current medication use.

### Statistical analysis

With respect to the statistical analyses of BMI, Hb concentrations, SBP, and DBP, the first measurements at study entry (gestational weeks 10 to <14) were used for the first trimester; the averages of measurements including the first antenatal visit at gestational weeks 14 to 16, around gestational weeks 20 and 24 were used for the second trimester; the averages of measurements around gestational weeks 32 and 36 were used for the third trimester. All analyses were performed using Stata, version 12 (StataCorp LP, College Station, Texas).

We fully evaluated the effects of high BMI and high Hb concentrations on blood pressure by trimesters of pregnancy. To do this, first, we generated two binary variables including *bmi* (dichotomized into high BMI and non-high BMI) and *hb* (dichotomized into high Hb and non-high Hb), and used multivariable linear regression to calculate the differences in SBP and DBP comparing high BMI or high Hb to the non-high category, respectively. Second, we used multivariable linear regression to evaluate the differences in SBP and DBP, comparing the combined categories of high BMI and high Hb, high BMI and non-high Hb, non-high BMI and high Hb to non-high BMI and non-high Hb, respectively. Third, using multivariable linear regression, we further assessed the interaction as departure from additivity between high BMI and high Hb on SBP and DBP by including *bmi*, *hb*, and the product of *bmi* and *hb* in models, and obtained the amount of interaction, which equaled the regression coefficient of the product term. The corresponding methodology was described by Knol et al. [16].

For multivariable regression, we fitted 3 models with progressive levels of adjustment. Model 1 was adjusted for age (continuous) and ethnicity (Han, Zhuang, other). Model 2 was further adjusted for education (<12 years, ≥12 years), family income < US$ 3000 (yes, no), urban residence (yes, no), parity (0, 1, >1), and folic acid supplement (yes, no). Model 3 was further adjusted for serum total cholesterol (continuous), serum triglycerides (continuous), and diabetes mellitus (yes, no).

## Results

The main characteristics of the study population by trimester are shown in Table 1. On average, the mean age of women by trimester ranged from 25.2 to 26.3 years. The means of BMI, Hb concentrations, SBP and DBP by trimester ranged from 22.9 to 26.5 kg/m^2^, 11.7 to 12.5 g/dL, 107.0 to 112.8 mmHg and 66.4 to 73.8 mmHg, respectively. BMI increased progressively during pregnancy, however, Hb concentrations, SBP and DBP in the second trimester were lower than in the first and third trimesters.

**Table 1:** **Baseline characteristics of the study population by trimesters of pregnancy**^**†**^

**Characteristic**	**1**^**st **^**trimester (N = 3472)**	**2**^**nd **^**trimester (N = 2986)**	**3**^**rd **^**trimester (N = 2261)**
Maternal age, year	26.0 (0.8)	26.3 (0.5)	25.2 (0.6)
The Han, n (%)	2226 (64.1)	1965 (65.8)	1526 (67.5)
Education (≥12 years), n (%)	1476 (42.5)	1170 (39.2)	1011 (44.7)
Family income (<$ 3000), n (%)	736 (21.2)	582 (19.5)	355 (15.7)
Urban residence, n (%)	1628 (46.9)	1582 (53.0)	1302 (57.6)
Parity (nulliparous), n (%)	2170 (62.5)	1765 (59.1)	1490 (65.9)
Folic acid supplement, n (%)	1430 (41.2)	1135 (38.0)	988 (43.7)
Serum total cholesterol, mg/dL	181.6 (1.1)	221.5 (0.7)	243.6 (0.8)
Serum triglyceride, mg/dL	124.9 (0.9)	177.1 (1.1)	256.8 (1.3)
Diabetes mellitus, n (%)	69 (2.0)	66 (2.2)	133 (5.9)
Body mass index, kg/m^2^	22.9 (0.2)	24.1 (0.2)	26.5 (0.3)
Hemoglobin, g/dL	12.5 (0.2)	11.7 (0.1)	11.8 (0.1)
Systolic blood pressure, mmHg	110.5 (1.3)	107.0 (1.1)	112.8 (1.0)
Diastolic blood pressure, mmHg	68.4 (0.6)	66.4 (0.5)	73.8 (0.7)

^†^Values are means (standard error) or number (percentage) unless otherwise noted.

**Figure 2:**
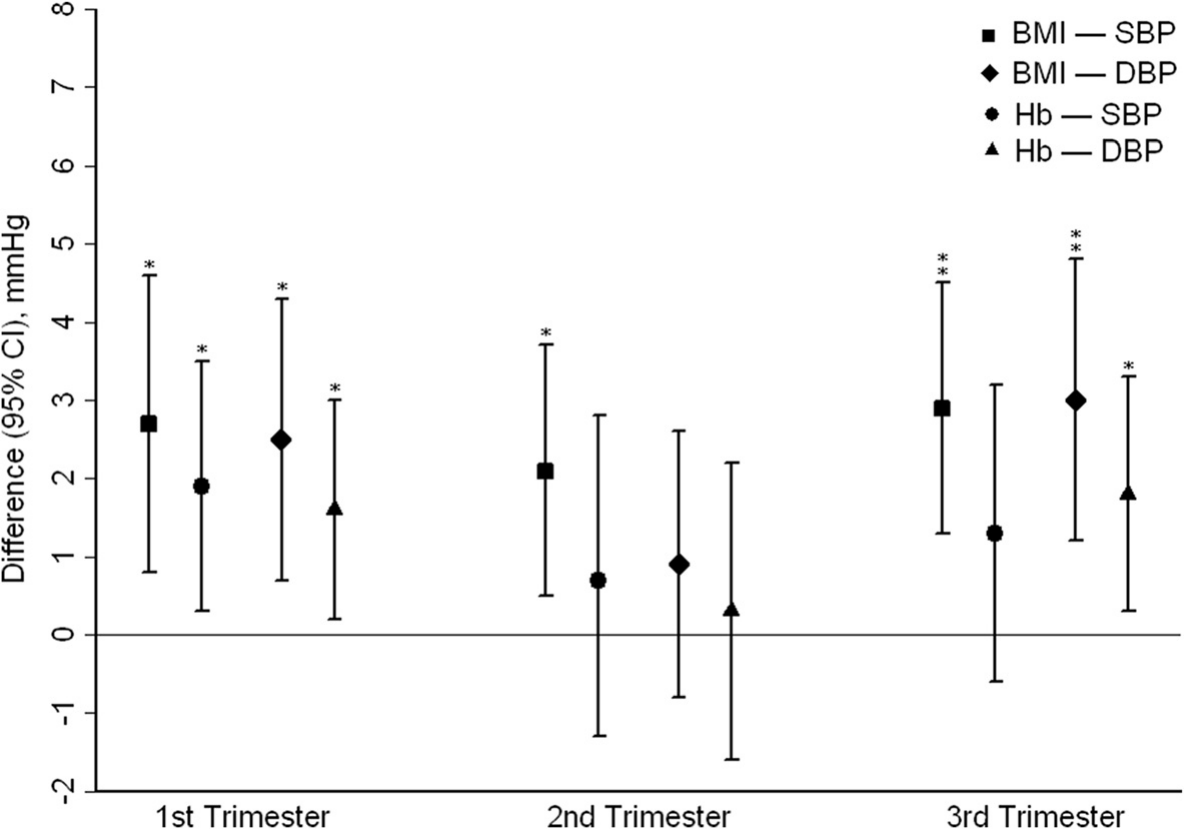
**Differences (95% CIs) in SBP and DBP with BMI and Hb by trimesters of pregnancy.** Differences were adjusted for age (continuous), ethnicity (Han, Zhuang, other), education (<12 years, ≥12 years), family income < US$ 3000 (yes, no), urban residence (yes, no), parity (0, 1, >1), folic acid supplement (yes, no), total cholesterol (continuous), triglycerides (continuous), and diabetes mellitus (yes, no). Comparing the high to the non-high categories: ^*^P <0.05, ^**^P <0.001.

In the first trimester, either of BMI and Hb was positively associated with both SBP and DBP (*P* <0.05) (Figure 2). In models adjusted for age and ethnicity, women with high BMI and high Hb had significantly increased differences for SBP and DBP compared to women with non-high BMI and non-high Hb. After full adjustment, however, the differences became weak, the average difference in women with high BMI and high Hb compared to women with non-high BMI and non-high Hb were 2.9 mmHg (95% CI: 0.8 to 5.0 mmHg) in SBP, and 3.9 mmHg (95% CI: 1.5 to 6.3 mmHg) in DBP, respectively (Table 2). We found no evidence of significant interaction between high BMI and high Hb on SBP (*P* = 0.14) and DBP (*P* = 0.38) in full-adjusted models (Table 3).

**Table 2:** Difference (95% CI) in blood pressure by categories of BMI and Hb levels in first trimester

	**Non-high BMI (<26.8 kg/m**^**2**^**)**		**High BMI (≥26.8 kg/m**^**2**^**)**	
	**Hb <13.0 g/dL**	**Hb ≥13.0 g/dL**	**Hb <13.0 g/dL**	**Hb ≥13.0 g/dL**
Number	2218	907	213	134
SBP (mmHg)^a^	109.7	111.7	112.4	113.3
Model 1^b^	0.0 (reference)	2.6 (0.9, 4.2)	3.0 (0.5, 5.5)	4.0 (2.1, 6.0)
Model 2^c^	0.0 (reference)	2.5 (0.8, 4.2)	2.9 (0.5, 5.3)	3.8 (1.7, 5.4)
Model 3^d^	0.0 (reference)	1.4 (-0.3, 3.1)	2.6 (0.3, 4.9)	2.9 (0.8, 5.0)
DBP (mmHg)^a^	67.5	69.0	70.0	72.4
Model 1^b^	0.0 (reference)	1.9 (-0.3, 4.2)	3.2 (0.6, 5.7)	5.1 (2.7, 7.6)
Model 2^c^	0.0 (reference)	1.9 (-0.2, 4.1)	3.2 (0.8, 5.6)	5.0 (2.7, 7.4)
Model 3^d^	0.0 (reference)	1.0 (-1.0, 3.0)	2.5 (0.1, 4.9)	3.9 (1.5, 6.3)

CI, confidence interval; SBP, systolic blood pressure; DBP, diastolic blood pressure; Hb, Hemoglobin.

^a^Mean SBP; mean DBP.

^b^Model 1: Adjusted for age (continuous) and ethnicity (Han, Zhuang, other).

^c^Model 2: Further adjusted for education (<12 years, ≥12 years), family income < US$ 3000 (yes, no), urban residence (yes, no), parity (0, 1, >1), and folic acid supplement (yes, no).

^d^Model 3: Further adjusted for total cholesterol (continuous), triglycerides (continuous), and diabetes mellitus (yes, no).

**Table 3:** **Regression coefficient (95% CI)**^**† **^**of additive model for blood pressure by categories of BMI and Hb levels in pregnancy**

**Variable**	**SBP**		**DBP**	
	***β *** **(95% CI)**	***P *** **value**	***β *** **(95% CI)**	***P *** **value**
**First trimester**				
Constant	70.3 (64.0, 76.6)	<0.001	55.6 (47.9, 63.3)	<0.001
*bmi*	2.6 (0.3, 4.9)	0.01	2.5 (0.1, 4.9)	0.03
*hb*	1.4 (-0.3, 3.1)	0.09	1.0 (-1.0, 3.0)	0.16
*bmi* × *hb*	-1.1 (-3.7, 1.6)	0.14	0.4 (-2.3, 3.1)	0.38
**Second trimester**				
Constant	72.1 (65.8, 78.4)	<0.001	56.9 (49.1, 64.7)	<0.001
*bmi*	1.9 (-0.5, 4.3)	0.22	0.8 (-1.5, 3.2)	0.32
*hb*	0.6 (-1.6, 2.8)	0.54	-0.2 (-2.3, 1.9)	0.63
*bmi* × *hb*	0.1 (-2.6, 2.8)	0.30	0.9 (-1.7, 3.5)	0.11
**Third trimester**				
Constant	74.1 (67.8, 80.4)	<0.001	59.3 (51.6, 67.0)	<0.001
*bmi*	2.0 (0.3, 3.7)	0.009	2.2 (0.2, 4.2)	0.01
*hb*	0.8 (-1.1, 2.7)	0.27	1.2 (-1.0, 3.4)	0.12
*bmi* × *hb*	2.0 (0.1, 3.9)	0.02	2.3 (0.4, 4.3)	0.004

^†^Adjusted for age (continuous), ethnicity (Han, Zhuang, other), education (<12 years, ≥12 years), family income < US$ 3000 (yes, no), urban residence (yes, no), parity (0, 1, >1), folic acid supplement (yes, no), total cholesterol (continuous), triglycerides (continuous), and diabetes mellitus (yes, no).

Only BMI was positively associated with SBP in the second trimester (*P* <0.05) (Figure 2). We further found significant differences in SBP comparing women with high BMI and high Hb to those with non-high BMI and non-high Hb, but non-significant in DBP regardless of multivariable-adjusted models. After full adjustment, the average differences in SBP and DBP, comparing women with high BMI and high Hb to those with non-high BMI and non-high Hb, were 2.6 mmHg (95% CI: 0.4 to 4.8 mmHg) and 1.5 mmHg (95% CI: -1.3 to 4.3 mmHg), respectively (Table 4). Also, there was no evidence of significant interaction between high BMI and high Hb on SBP (*P* = 0.3) or DBP (*P* = 0.11) in full-adjusted model (Table 3).

**Table 4:** Difference (95% CI) in blood pressure by categories of BMI and Hb levels in second trimester

	**Non-high BMI (<28.5 kg/m**^**2**^**)**		**High BMI (≥28.5 kg/m**^**2**^**)**	
	**Hb <13.0 g/dL**	**Hb ≥13.0 g/dL**	**Hb <13.0 g/dL**	**Hb ≥13.0 g/dL**
Number	2201	488	190	109
SBP (mmHg)^a^	106.3	107.7	108.6	108.9
Model 1^b^	0.0 (reference)	1.1 (-1.3, 3.4)	2.2 (-0.6, 5.0)	3.2 (1.0, 5.5)
Model 2^c^	0.0 (reference)	0.9 (-1.5, 3.3)	2.1 (-0.4, 4.6)	3.0 (0.9, 5.1)
Model 3^d^	0.0 (reference)	0.6 (-1.6, 2.8)	1.9 (-0.5, 4.3)	2.6 (0.4, 4.8)
DBP (mmHg)^a^	66.2	66.4	67.2	68.1
Model 1^b^	0.0 (reference)	0.5 (-1.9, 2.8)	1.4 (-1.0, 3.8)	2.2 (-0.8, 5.2)
Model 2^c^	0.0 (reference)	0.2 (-2.0, 2.5)	1.2 (-1.1, 3.7)	2.2 (-0.7, 5.1)
Model 3^d^	0.0 (reference)	-0.2 (-2.3, 1.9)	0.8 (-1.5, 3.2)	1.5 (-1.3, 4.3)

CI, confidence interval; SBP, systolic blood pressure; DBP, diastolic blood pressure; Hb, Hemoglobin.

^a^Mean SBP; mean DBP.

^b^Model 1: Adjusted for age (continuous) and ethnicity (Han, Zhuang, other).

^c^Model 2: Further adjusted for education (<12 years, ≥12 years), family income < US$ 3000 (yes, no), urban residence (yes, no), parity (0, 1, >1), and folic acid supplement (yes, no).

^d^Model 3: Further adjusted for total cholesterol (continuous), triglycerides (continuous), and diabetes mellitus (yes, no).

In the third trimester, BMI was positively with both SBP and DBP, but higher Hb was only associated with higher DBP (*P* <0.05) (Figure 2). After adjustment for age and ethnicity, there were significant increases in the differences for SBP and DBP compared to women with non-high BMI and non-high Hb. Full adjustment for confounding did not materially affect this association, the average differences in SBP and DBP, comparing women with high BMI and high Hb to those with non-high BMI and non-high Hb, were 4.8 mmHg (95% CI: 2.3 to 7.4 mmHg) and 5.7 mmHg (95% CI: 3.2 to 8.3 mmHg), respectively (Table 5). For the interaction, we found significant synergistic effects between high BMI and high Hb on SBP (*P* = 0.02) and DBP (*P* = 0.004) in full-adjusted models, the amount of interaction on SBP and DBP were 2.0 mmHg (95% CI: 0.1 to 3.9 mmHg) and 2.3 mmHg (95% CI: 0.4 to 4.3 mmHg), respectively (Table 3).

**Table 5:** Difference (95% CI) in blood pressure by categories of BMI and Hb levels in third trimester

	**Non-high BMI (<30.9 kg/m**^**2**^**)**		**High BMI (≥30.9 kg/m**^**2**^**)**	
	**Hb <13.0 g/dL**	**Hb ≥13.0 g/dL**	**Hb <13.0 g/dL**	**Hb ≥13.0 g/dL**
Number	1435	600	148	78
SBP (mmHg)^a^	111.3	112.5	113.6	116.9
Model 1^b^	0.0 (reference)	1.3 (-1.0, 3.7)	2.2 (0.3, 4.1)	5.8 (3.2, 8.4)
Model 2^c^	0.0 (reference)	1.2 (-1.0, 3.4)	2.2 (0.2, 4.1)	5.6 (3.1, 8.1)
Model 3^d^	0.0 (reference)	0.8 (-1.1, 2.7)	2.0 (0.3, 3.7)	4.8 (2.3, 7.4)
DBP (mmHg)^a^	72.5	74.0	74.8	78.5
Model 1^b^	0.0 (reference)	1.9 (-0.6, 4.3)	2.5 (0.4, 4.7)	6.5 (3.8, 9.1)
Model 2^c^	0.0 (reference)	1.7 (-0.5, 3.9)	2.4 (0.3, 4.5)	6.2 (3.6, 8.8)
Model 3^d^	0.0 (reference)	1.2 (-1.0, 3.4)	2.2 (0.2, 4.2)	5.7 (3.2, 8.3)

CI, confidence interval; SBP, systolic blood pressure; DBP, diastolic blood pressure; Hb, Hemoglobin.

^a^Mean SBP; mean DBP.

^b^Model 1: Adjusted for age (continuous) and ethnicity (Han, Zhuang, other).

^c^Model 2: Further adjusted for education (<12 years, ≥12 years), family income < US$ 3000 (yes, no), urban residence (yes, no), parity (0, 1, >1), and folic acid supplement (yes, no).

^d^Model 3: Further adjusted for total cholesterol (continuous), triglycerides (continuous), and diabetes mellitus (yes, no).

## Discussion

In this study, differences in the associations of BMI and Hb with either SBP or DBP were present throughout pregnancy. BMI was positively associated with SBP across 3 trimesters, but with DBP in the first and third trimesters; higher Hb concentrations were associated with increased SBP only in the first trimester, but with increased DBP in the first and third trimesters. Furthermore, we found significant interaction between high BMI and high Hb on elevated both SBP and DBP only in the third trimester.

Some observational studies have consistently shown a positive association between maternal BMI and blood pressure, whether in pregnancy [3-6] or prepregnancy [17-20], although these associations were concluded by different statistical models. A recent system review including 13 trials, however, indicates that dietary intervention appears effective to reduce total and weekly gestational weight gain, but no significant effect on preventing preeclampsia and gestational diabetes [21]. For Hb, several observational studies found significantly increased Hb concentrations in women who developed PIH in the second or third trimester [7-9]. Also, a recent study showed that Hb level was positively associated with both SBP and DBP in a large cohort of healthy individuals [22]. In our analysis, we excluded women with PIH or preeclampsia mainly owing to antihypertensive medication, in contrast to previous research that defined outcome as PIH or preeclampsia, our study thus focused on the physiological effects of BMI and Hb on blood pressure in the whole range of trimesters of pregnancy. As a whole, our finding regarding the positive associations of BMI and Hb with blood pressure supports previous research.

The mechanisms for increased BMI or Hb concentrations with elevated blood pressure in pregnancy have been postulated. For maternal BMI, the associations of gestational adiposity with dyslipidaemia, hyperglycaemia, and insulin resistance are well recognized [4,23-25]. These risk patterns lead to oxidative stress and lipoprotein oxidation contributing to endothelial dysfunction [26] that is characterized by the pathogenesis of preeclampsia [27]. Additionally, several functional changes associated with maternal obesity, including lipid alternations, sleep apnea, and increased cardiac output and oxygen consumption, are involved in hypertensive disorders in pregnancy [28-30]. For Hb, the mechanisms that contribute to hypertensive disorders of pregnancy may be related with blood viscosity, which can be induced by elevation of hematocrit and Hb levels, since it has been suggested that increased blood viscosity may worsen cardiovascular function partly through an effect on blood pressure [31]. Given this finding regarding blood viscosity, it might probably explain the previous studies [7-9] that the positive associations between increased Hb and PIH were in agreement with elevated blood viscosity during pregnancy. Furthermore, free Hb is a scavenger of nitric oxide (NO), which can relax the muscle cells, increased free Hb levels may thus bind to NO and contribute to vasoconstriction and hypertension [32,33]. With so many potential mechanisms involved, perhaps it is not surprised by the differences in the associations of BMI and Hb concentrations with SBP or DBP throughout pregnancy.

This is the first analysis to our knowledge of the potential interaction between high BMI and high Hb concentrations on blood pressure in pregnancy. Our main finding is that both SBP and DBP are significantly elevated in women with high BMI and high Hb in the third trimester of pregnancy, and the interaction between high BMI and high Hb indicates a synergistic effect on blood pressure. In contrast to the second trimester, both high BMI and high Hb were more likely to raise higher blood pressure in the first and third trimesters, however, further comparison with the first trimester, blood pressure elevated by the individual effect of high BMI or high Hb was consistent in the third trimester. Therefore, this suggests that the interaction in the third trimester, may not only depend on the excess amounts in blood pressure raised by high levels of BMI and Hb, but also the physiological changes in pregnancy. In the present analysis, even although the synergistic effects on SBP and DBP increased only 2 mmHg and 2.3 mmHg, respectively, perhaps we cannot rule out the possible adverse effects on pregnancy outcomes. Randomized controlled trial data have shown that lowering SBP of 3 mmHg may result in a 16% reduction of cardiovascular events and a 22% reduction of fatal and nonfatal strokes [34]. For pregnant women, the biological effect of interaction on increased blood pressure, even with small scale, thus needs to be confirmed in further studies. Moreover, the tendency of blood pressure in women with high BMI and high Hb concentrations may need to be monitored with care during the antenatal visit.

The mechanisms underlying the interaction between high BMI and high Hb on increased blood pressure are unclear. Based on the association of elevated blood viscosity with adiposity or increased Hb levels [7,10-13], the present study partly supports our hypothesis that high BMI and high Hb may have a combined effect on increased blood pressure via elevated blood viscosity during normal pregnancy. However, we do not have a good explanation on mechanism for the interaction in the third trimester, but not in the first and second trimesters. Generally in normal pregnancy, blood volume, stroke volume, and cardiac output increase progressively from the first trimester until term [35], whereas blood viscosity has a drop until 29 weeks gestation, followed by a small increase toward term [36]. In our study, we cannot determine the change in hemodynamics owing to lack of measuring blood viscosity using viscometer, further studies thus need to systematically evaluate the role of blood viscosity in the associations of increased BMI and Hb concentrations with blood pressure in pregnancy.

The present study has some limitations. First, owing to our cross-sectional analysis, we cannot determine the causality between increased BMI or Hb concentrations and elevated blood pressure. Second, the physiological adaptations in maternal BMI, Hb concentrations, and blood pressure are dynamic throughout pregnancy. We cannot collect blood samples at every antenatal visit due to the schedule in routine antenatal care, hence this probably weakened the representation of 3 trimesters of pregnancy only using the measurement at study entry, around gestational weeks 20 and 24, 32 and 36. Third, we accumulatively lost 943 (21%) and 417 (14%) participants with the follow-up in two subsequent trimesters, respectively. Based on our analysis, the characteristics of participants lost to follow up were not significantly different with participants who remained in follow up except for family income and urban residence. Moreover, the regression coefficients in each trimester did not significantly change after adjustment for the two variables, our results thus did not substantially be influenced by loss to follow up.

## Conclusions

BMI and Hb concentrations appear to have different associations with blood pressure throughout pregnancy. Even more important, our findings indicate a synergistic effect between high BMI and high Hb on blood pressure in the third trimester of pregnancy. Further prospective research is needed to identify the biological mechanism of interaction, and evaluate its clinical role in pregnancy.

## Competing interests

The authors declare that they have no competing interests.

## Authors’ contributions

QZ, JX and XQ designed the research and wrote the manuscript. QZ and YD analyzed the data. YL contributed in the program development and participated in the program protocol. JH and XL collaborated in the data interpretation and assisted in data analysis. All authors reviewed and edited manuscript. The final manuscript was read and approved by all authors.

## Pre-publication history

The pre-publication history for this paper can be accessed here:

http://www.biomedcentral.com/1471-2458/14/474/prepub
